# Two rare case reports of familial cyclic neutropenia caused by ELANE gene mutation

**DOI:** 10.1097/MD.0000000000042201

**Published:** 2026-01-16

**Authors:** Kai Wang, Wenfeng Huang, Jihong Zhu

**Affiliations:** a Department of Emergency, Peking University People’s Hospital, Beijing, China.

**Keywords:** autosomal dominant inheritance, case report, ELANE gene mutation, familial cyclic neutropenia

## Abstract

**Rationale:**

This article presents 2 exceptionally rare cases of familial cyclic neutropenia (CN), along with a comprehensive review of the relevant literature. It further explores the clinical manifestations, laboratory examination characteristics, diagnostic approaches, and treatment strategies, as well as prognosis associated with CN. The aim of this paper is to enhance clinicians’ awareness of these rare diseases, thereby facilitating early diagnosis and appropriate management.

**Patient concerns:**

Case 1: a 47-year-old female who had suffered from bacillary dysentery, recurrent rectal abscess, anal fistula, gingivitis, and periodontitis from the age of 28, otherwise she had suffered from upper respiratory tract infection twice or 3 times every year with neutrophil count nadir of 0.18 × 10^9^/L. Exome sequencing of blood of the woman and her family revealed this woman and her 7 years old daughter had the same c.416C > T(p.P139L) heterozygous mutation in the ELANE gene. Case 2: this patient was the daughter of case 1. The patient developed bronchial infection from the age of 2, and the blood routine showed the neutrophils were abnormally low. In addition, repeated cough, sputum and fever occurred during the year, and neut decreased in 7 of the 11 routine blood tests. The result of exome sequencing of blood revealed that she had the same c.416C > T(p.P139L) heterozygous mutation in the ELANE gene.

**Diagnoses:**

The 2 patients were diagnosed with familial CN.

**Interventions:**

Intermittent Chinese medicine, pidotimod, mannanopeptide treatment, and symptomatic treatment.

**Outcomes:**

A rare case of familial transmission involving 2 instances of mother–daughter pairs. Blood exome sequencing revealed that both pairs harbored the identical heterozygous mutation c.416C > T(p.P139L) in the ELANE gene, leading to a definitive diagnosis of familial CN caused by this ELANE gene mutation. At present, the patients’ conditions are improved and the outpatient clinic is followed up regularly.

**Lessons:**

If the patient presents with recurrent infections, fever, and other periodic symptoms annually, accompanied by neutropenia as indicated by a complete blood count, CN should be considered as a potential diagnosis. Genetic testing is imperative to ascertain the presence of any gene mutations for an accurate diagnosis. Cyclic neutropenia can be diagnosed based on the patient’s clinical manifestations, laboratory tests, and genetic testing results.

## 1. Introduction

Cyclic neutropenia (CN) is a rare disease associated with neutrophil production. The frequency of this disorder is estimated to be 1 per 1,000,000 in US metropolitan areas. The disease is characterized by fever, aphthous stomatitis, lymphadenopathy, and infections resulting from neutropenia that recurs every 14 to 28 days.^[1]^ Some individuals display cycling of other blood cells with the same periodicity. These manifestations diminish with age. CN can present as a sporadic, congenital, acquired or as an autosomal dominantly inherited disorder. At present, the disease has been confirmed to be associated with a variety of gene mutations, mainly occurring in genes such as ELANE, HAX1, GFI-1, WASP, G6PC3, CSF3R, and JAGN1, of which 50% to 60% of cases are caused by ELANE gene mutations.^[2,3]^

Due to the clinical heterogeneity and low incidence of CN, missed and inaccurate diagnosis are common, resulting in poor prognosis. Here, we describe 2 females in a family with CN. Chromosomal analysis shows a pericentric inversion of the ELANE gene mutation. Written informed consent for case reports was obtained from patients or their relatives. This study was conducted in accordance with the guidelines outlined in the Declaration of Helsinki. Nevertheless, this study has certain limitations. The genetic tracking and research were not conducted systematically across the entire family lineage of the 2 familial cases; instead, the investigation was confined solely to the mother–daughter dyads. Furthermore, additional longitudinal follow-up studies are warranted. Additionally, this study lacks a continuous follow-up investigation regarding the prognosis of the studied cases. The relevant information of the 2 patients was reported through their personal/legal guardian consent and signed written informed consent. This research received approval from the Ethics Committee of Peking University People’s Hospital (No. 2024PHB066-001). The relevant information of the 2 patients was reported through their personal/legal guardian consent and signed written informed consent. The datasets generated during and/or analyzed during the current study are available from the corresponding author on reasonable request.

## 2. Case 1

**Figure 1.**
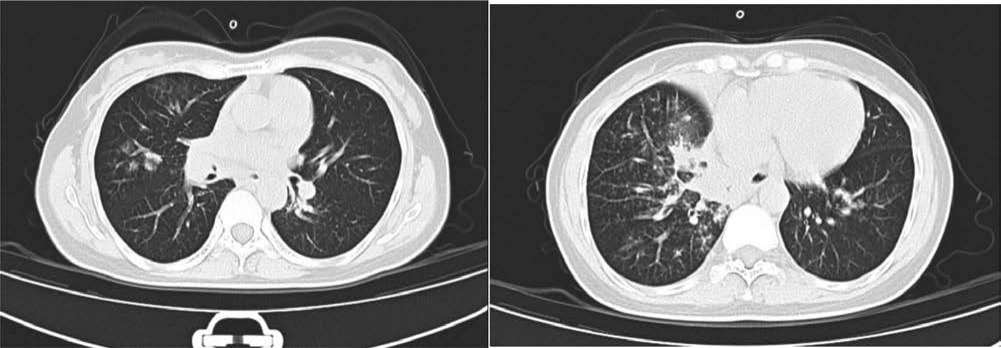
Chest CT showed bilateral multiple patchy high-density shadows, multiple bronchial wall thickening in both lungs, partial lumen stenosis, and local lamellar consolidation in the lower lobe of the right lung. Multiple lymph nodes are seen in the mediastinum; multiple infections in both lungs, local atelectasis in the right lung, multiple bronchial mucus plugs in both lungs. Multiple mediastinal lymph nodes.

The patient was a 47-year-old female who was admitted to the hospital because of “cough, purulent sputum, and fever for 5 days.” Five days before admission, the patient developed cough and sputum symptoms after exhaustion and anxiety, accompanied by fever, fatigue, and dyspnea. Her temperature was up to 39.2 ℃, the other vital signs were within normal limits. Both lungs had coarse breathing sounds, the right lung had obvious moist rales, and the other physical examination was unremarkable. Her laboratory test results: white blood count 4.4 to 2.56 × 10^9^/L, neutrophil count 1.74 to 0.46 × 10^9^/L, C-reactive protein 7 to 195.3 mg/L, procalcitonin 0.125 ng/mL, the blood gas was basically normal (pH: 7.5, pCO_2_: 30 mm Hg, pO_2_: 79 mm Hg, HCO_3_: 23.4 mmol/L), the liver and renal functions were normal. Serological tests for hepatitis B, C, and HIV were negative. She had an elevated IgG level of 28.92 g/L, while IgA, IgM, and IgG subclasses were within normal ranges. Sputum smear revealed the presence of 2 + Gram-positive cocci and 2 + Gram-negative cocci, whereas sputum culture was sterile. Chest CT showed infection and bronchial mucus plugs of bilateral pulmonary and the local atelectasis in the right lung (Fig. 1). The bronchoscopy indicated congestion and edema of the mucosa in the bilateral main bronchus and distal lobar bronchus, particularly in the right middle and lower lobar bronchus, stenosis of the right middle lobe bronchial lumen, with mild stenosis in the remaining lumen and there is slightly more white secretion in the lumen. The bronchoalveolar lavage fluid (BALF) smear revealed the presence of 1 + Gram-positive cocci, the galactomannan assay of BALF was positive, the next-generation sequencing of BALF showed positive results for human herpesvirus 1 and *Haemophilus influenzae*, and suspected positive result for human metapneumovirus.

**Figure 2.**
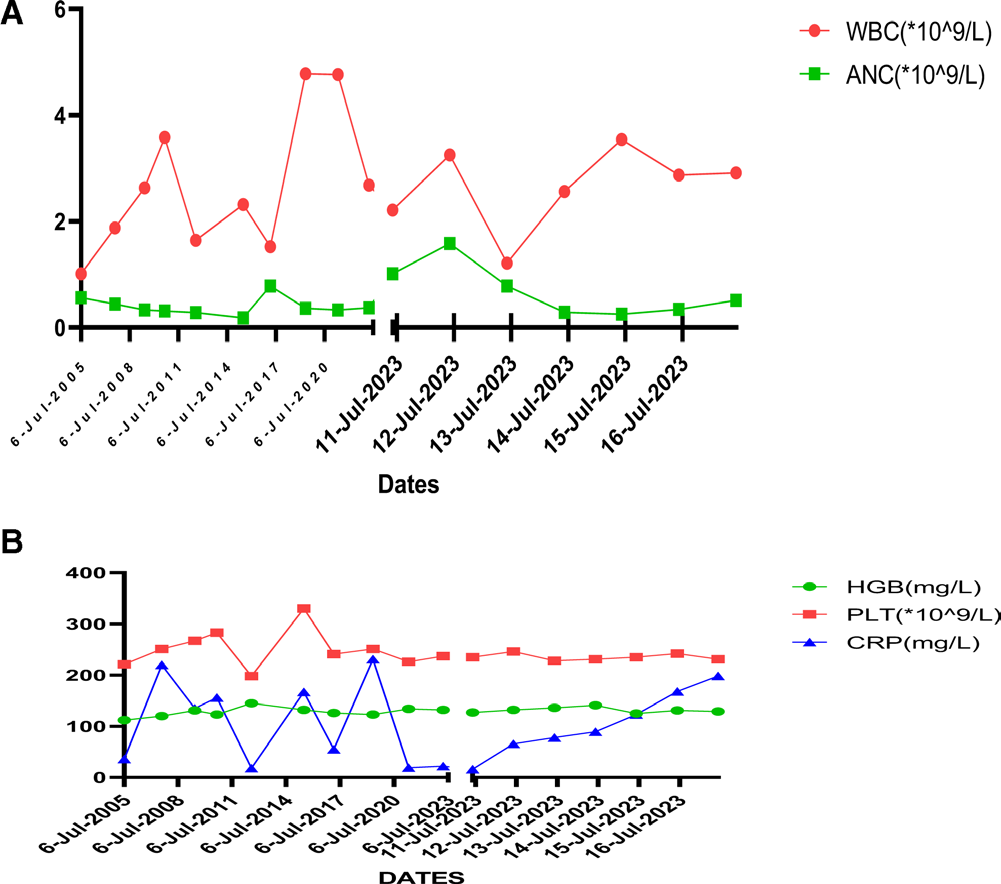
(A) Results of WBC, ANC, and Hb testing at different times; (B) results of PLT and CRP testing at different times. ANC = absolute neutrophil count, CRP = C-reactive protein, HGB = hemoglobin, WBC = white blood cells.

Medical history showed that she had suffered from bacillary dysentery, recurrent rectal abscess, anal fistula, gingivitis, and periodontitis from the age of 28, otherwise she had suffered from upper respiratory tract infection twice or 3 times every year with neutrophil count nadir of 0.18 × 10^9^/L (Fig. 2). Exome sequencing of blood of the woman and her family revealed this woman and her 7 years old daughter had the same c.416C > T(p.P139L) heterozygous mutation in the ELANE gene, but her husband had not carried the mutation (electropherograms were not available). This suggests that the mutated gene came from the patient himself and was passed on to his daughter through autosomal dominance (Table 1).

**Table 1:** Results of genetic testing in the patient’s family.

Gene	Chromosomal location	Transcription number	Exon	Nucleotide change	Amino acid change	Homozygous/heterozygous	Pathogenicity analysis	Mode of inheritance	Disease/phenotype	Source of variation
ELANE	chr19-855613	NM_001972	Exon 4	c.416C>T	p.P139l	het	Likely_pathogenic	AD	SCN/CN	Mother

Gene sequencing results: the child had an ELANE mutation C.416C > T (exon 4), while the parents of the child had normal wild type ELANE without heterozygous variation. The ELANE missense mutation C.416C > T (exon 4), located on chromosome 19 (19 p.P139l).

AD = autosomal dominant inheritable disease, CN = cyclic neutropenia, SCN = severe cyclic neutropenia.

## 3. Case 2

**Figure 3.**
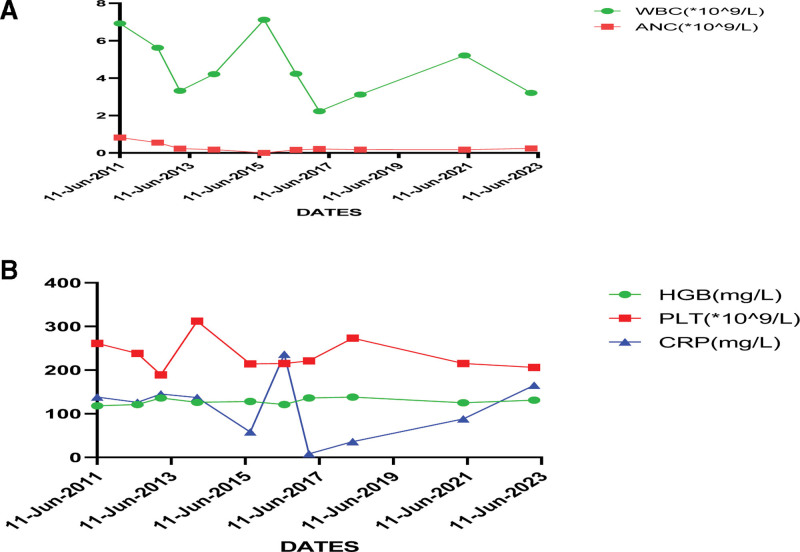
(A) Results of WBC, ANC, and Hb testing at different times; (B) results of PLT and CRP testing at different times. ANC = absolute neutrophil count, CRP = C-reactive protein, HGB = hemoglobin, WBC = white blood cells.

This patient was the daughter of the first patient. The patient developed bronchial infection from the age of 2, and the blood routine showed white blood cells 6.92 to 5.62 × 10^9^/L, NEUT 0.82 to 0.55 × 10^9^/L, hemoglobin 117 g/L, PLT 347 × 10^9^/L. In addition, repeated cough, sputum and fever occurred during the year, and neut decreased in 7 of the 11 routine blood tests. Intermittent Chinese medicine, pidotimod, mannanopeptide treatment. The patient began to develop recurrent gingivitis and periodontitis at the age of 3, gum swelling with oral ulcers, and in severe cases with pharyngeal pain, sinusitis, and otitis media. Respiratory tract infection occurred again at the age of 5, and blood routine showed white blood cells 7.24 × 10^9^/L, neut 0 × 10^9^/L, Lym 3.12 × 10^9^/L, hemoglobin 118 g/L, PLT 308 × 10^9^/L, C-reactive protein 116 mg/L, repeated oral ulcers (Fig. 3). No joint swelling pain, photoallergy, erythema, with “infectious mononucleosis” admitted. Bone marrow aspiration: bone marrow images: active bone marrow hyperplasia, poor grain system hyperplasia, increased cytoplasmic particles in the middle and young particles, toxic particles in mature cells. The proportion of red series is reduced, mainly medium and young red, the ratio of grain red is low and the shape is generally normal. Increased megakaryocyte and platelet ratio. The increase of mature monocytes. Appendicitis developed at the age of 7, and the appendix became infected with an abscess. The exome sequencing of blood of the child and her parents was performed, the results revealed this child and her mother had the same c.416C > T(p.P139L) heterozygous mutation in the ELANE gene, but her father had not carried the mutation (electropherograms were not available). The final diagnosis was considered periodic neutropenia, a mutation derived from the mother and currently passed on to the patient via autosomal dominant.

## 4. Discussion

The pathogenic genes of CN include EIANE, GFll, HAXl, SBDS, etc. The most common pathogenic gene is EIANE mutation, which is inherited by single gene autosomal dominant inheritance. Bone marrow cell differentiation stops at an early stage and cannot produce normal function or number of mature neutrosomes. According to the variation of absolute neutrophil count and the difference of clinical symptoms, the phenotypes of EIANE mutations are SCN and CN. Currently, far more patients with SCN than CN have been reported.^[4,5]^ We have reported the above cases that the mother had cyclic neutropenia caused by ELANE gene mutation, and then passed it on to her daughter through autosomal dominance. It is very rare for a mother to have a genetic mutation and pass it on to her daughter. We searched Pubmed, Web of Science and no similar case was reported. The specific mechanism by which EIANE mutations ultimately cause neutropenia by affecting the structure or function of neutrophil elastase is unknown, the most common being the unfolded protein response theory. Both SCN and CN patients present with early onset, repeated respiratory tract infection, skin and mucous membrane infection, and severe infections can be life-threatening and malignant transformation into myelodysplastic syndrome or acute myeloid leukemia. It is an important cause of poor prognosis.^[5]^

Regarding the pathogenesis of EIANE gene mutation, it is necessary to understand the structure of EIANE gene firstly. At present, there are more than 200 EIANE gene mutations in the ClinVar database, including missense mutations, nonsense mutations, synonymous mutations, frameshift mutations, intron shear, and other forms, mainly missense mutations, sporadic mutations, and familial genetic cases have not been significantly different in the number of reported cases.^[6,7]^ The mutation sites include not only the coding region of the mature enzyme, but also the proenzyme region and the shoulder motor region, and the mutation sites tend to be near the active site of the enzyme (exons 3, 4, 5 and introns 3, 4) 110. Both SCN and CN cases of A57V, G97P, S126L, P139L, V190 F199 del, and W241X have been reported in the literature. The same genotype has 2 different clinical phenotypes, and the specific mechanism is still unclear. EIANE mutations such as V65del, N116D, C1 51Y, G185R, P205R, G214R, G214V, both SCN and CN, tend to exhibit more severe clinical symptoms and develop myelodysplastic syndrome or acute myeloid leukemia. Or a higher risk of death.^[7,8]^ Two-site mutations in CN cases tended to show more severe clinical symptoms, such as A233P with V235wfsx mutation and G192A with 193 195del mutation.^[9]^

Neutrophils make up the body’s infection-fighting immune system, and severe neutropenia tends to cause serious recurrent bacterial or fungal infections, with *Staphylococcus aureus*, *Pseudomonas aeruginosa*, and *Escherichia coli* being the most common.^[10]^ The aforementioned cases have indeed exhibited recurrent bacterial and fungal infections, involving pathogens such as *Streptococcus pneumoniae*, *S aureus*, *Klebsiella pneumoniae*, and the fungus *Candida albicans*. The onset age of EIANE mutation-related SCN patients is usually <6 months after birth, and CN patients are mostly around 1 year old. Most CN patients have stomatitis as the first clinical symptom, while SCN patients often have omphalitis, stomatitis, or pneumonia as the first clinical symptom. Our cases the daughter developed the disease when she was about 2 years old. The first symptom of the disease is bronchitis, a respiratory infection. Most SCN patients suffer from lifelong recurrent infection, and the incidence of CN is lower than that of SCN, which is manifested by periodic decrease in the number of neutrophils, with a period of 14 to 36 days (about 21 days in most cases) lasting for about 3 to 7 days, and clinical symptoms also show periodic changes. In both cases, respiratory system and skin mucosa are the most common infection sites. In 2015, MAKARYAN et al^[5]^ reported that 307 patients with EIANE mutation-related neutropenia were infected. Common infections were oral ulcers (80%), pneumonia (49%), abscesses (19%), septicemia (17%), cellulitis (12%), and peritonitis (3%). Respiratory infections in CN patients are mostly concentrated in the oropharynx, and recurrent oral ulcers are the main symptoms, while oropharyngeal infections in SCN patients are more commonly manifested as periodontitis, including gingivitis, loose teeth, gingival redness, gingival bleeding, alveolar bone resorption, etc. Chronic injury may lead to early gingival necrosis and tooth loss. The frequency of lower respiratory tract infection in SCN is higher than that in CN. The onset of lung infection is early and easy to repeat. Some patients have trachea and bronchial dysplasia or pulmonary fibrosis. The MAKARYAN et al^[5]^ study reported that 63% of 97 patients with SCN developed pneumonia, compared with 19% of 26 patients with CN. Respiratory failure may occur in severe infections, requiring oxygen inhalation or even intubation to maintain oxygen saturation. The skin and mucous membrane infections of SCN and SCN are usually manifested as abscesses with no or little pus.^[11,12]^ The common infection sites of SCN are perianal and umbilical cord, and surgical treatment is required in severe cases. In addition, otitis media, mastoiditis, lymphadenitis, bone mass loss, anemia are also often reported.^[13–15]^

Prevention of infection contributes to long-term management of patients, G-CSF medication helps to improve clinical symptoms, and HSCT is currently the only treatment used to cure the disease. NE inhibitors are still at a very early stage as an alternative therapy for G-CSF, and gene editing is also a research direction for future therapies.^[16–18]^

## Author contributions

**Data curation:** Kai Wang.

**Formal analysis:** Jihong Zhu.

**Resources:** Wenfeng Huang, Jihong Zhu.

**Writing – original draft:** Kai Wang.

**Writing – review & editing:** Kai Wang, Wenfeng Huang, Jihong Zhu.
